# Rheology of Suspensions of TEMPO-Oxidised and Cationic Cellulose Nanofibrils—The Effect of Chemical Pre-Treatment

**DOI:** 10.3390/gels10060367

**Published:** 2024-05-26

**Authors:** Luís Alves, Solange Magalhães, Jorge F. S. Pedrosa, Paulo J. T. Ferreira, José A. F. Gamelas, Maria Graça Rasteiro

**Affiliations:** University of Coimbra, CERES, Department of Chemical Engineering, 3030-790 Coimbra, Portugal; solangemagalhaes@eq.uc.pt (S.M.); jpedrosa@uc.pt (J.F.S.P.); paulo@eq.uc.pt (P.J.T.F.); jafgas@eq.uc.pt (J.A.F.G.); mgr@eq.uc.pt (M.G.R.)

**Keywords:** hydrogels, cellulose nanofibres, degree of polymerization, charge density, entanglement, aggregation

## Abstract

Cellulose nanofibrils (CNFs) are particles with a high aspect ratio. Typically, chemically pre-treated CNFs (containing anionic or cationic charged groups) consist of long fibrils (up to 2 μm) with very low thickness (less than 10 nm). Derived from their high aspect ratio, CNFs form strong hydrogels with high elasticity at low concentrations. Thus, CNF suspensions appear as an interesting rheology modifier to be applied in cosmetics, paints, foods, and as a mineral suspending agent, among other applications. The high viscosity results from the strong 3D fibril network, which is related to the good fibrillation of the material, allowing the nanofibrils to overlap. The overlap concentration (c*) was found to vary from ca. 0.13 to ca. 0.60 wt.% depending on the type and intensity of the pre-treatment applied during the preparation of the CNFs. The results confirm the higher tendency for the fibres treated with (3-chloro-2-hydroxypropyl) trimethylammonium chloride (CHPTAC) and 2,2,6,6-tetramethylpiperidine 1-oxyl (TEMPO) to form a 3D network, resulting in the lowest c*. For the TEMPO-oxidised CNF suspensions, it was also found that aggregation is improved at acidic pH conditions due to lower charge repulsion among fibrils, leading to an increase in the suspension viscosity as well as higher apparent yield stresses. TEMPO CNF suspensions with a low content of carboxylic groups tend to precipitate at moderately acidic pH values.

## 1. Introduction

Cellulose micro/nanofibres are a quite recent type of nanomaterial that can be obtained by intensive mechanical treatment of cellulose fibres. The most common mechanical treatments are high-pressure homogenization (HPH), microfluidization, grinding, twin screw extrusion, cryo-crushing, and high-intensity ultrasonication [1,2]. Usually, the cellulose fibres treated only by mechanical processes do not present a high level of conversion to nanofibres, even when a highly intensive treatment is applied; for example, cellulose fibres treated only by mechanical treatment using HPH with two passes only resulted in ca. 6% conversion to nanofibres [3]. Even when increasing the number of cycles, the yield in nanofibres is usually low; for example, Jeencham et al. reported a yield in nanofibers of 14.7% when applying 15 cycles of HPH when preparing CNF from cassava pulp [4].

The chemical pre-treatment is a suitable way to improve the nanofibrillation degree. Among the chemical pre-treatments commonly used, TEMPO-mediated oxidation is one of the most widespread. The nanofibres obtained from TEMPO-oxidised fibres consist of very thin (less than 10 nm) and long fibrils, which result in particles with a high aspect ratio [5]. The high aspect ratio and the crystallinity are key factors for the formation of stiff hydrogels at relatively low CNF concentrations [6]. This favourable property enables the use of CNFs as rheology modifiers with potential applications in paints, foods, cosmetics, and suspending agents in mineral applications, among others [7].

Another pre-treatment suitable for the preparation of CNFs that has emerged recently is cationization [8]. The introduction of cationic groups, especially quaternary ammonium groups into the cellulose structure not only enhances the degree of fibrillation but also expands the types of applications due to the new CNF properties introduced by the cationic groups, such as antimicrobial properties [9]. Another recent application that presents high potential is the use of cationic CNFs as flocculants in wastewater treatment [10]. The introduction of positively charged groups can be achieved by different processes, with the most common being direct cationization through the reaction of the cellulose fibres with 2,3-epoxypropyltrimethylammonium chloride (EPTAC) [11], and a two-step cationization, using Girard’s reagent T (GT) after oxidation by periodate [8]. The first method, due to the nature of the modification (direct cationization in mild conditions), does not result in extended depolymerization of cellulose; contrary to this, the second method, in which strong oxidation is applied to form cellulose dialdehyde, being the cationic groups added in aldehyde moieties, results in large depolymerization of cellulose. On the other hand, the amount of cationic groups that can be introduced using the first method is quite limited, but for the second method, high-charge density CNFs can be obtained [8]. As expected, the fibrillation degree obtained when applying the first or second method is quite different, with the second method (for highly charged celluloses) being able to produce nanofibres with a larger extension.

The charge density, the fibrillation degree, and the fibril dimensions have a deep impact on the rheology of CNF suspensions [6] because the rheology of polymeric solutions or CNF suspensions is controlled by the physical entanglement of polymeric chains/cellulose nanofibres and their aggregation [12,13]. Nechyporchuk et al. pointed out that the CNF suspensions produced using mechanical fibrillation, with or without enzymatic pre-treatment (no surface chemical modification), possess highly flocculated structure and are not able to induce a high increase in the rheology of suspensions, contrary to the ones containing surface modifications, such as carboxylation, carboxymethylation, and quaternization [14]. In the case of CNFs containing charged groups, in which the charge density could be affected by external stimulus, as it is the case of carboxylic groups in TEMPO CNFs, it is possible to tune the rheology of the suspensions by changing the pH conditions [13,15,16]. In the literature are reported works dealing with the study of different ways to control the rheology of TEMPO CNFs, proposing the addition of salts [15,16,17], the ageing effect [18], gelation induced by heat [19], gelation induced by alcohol addition [20], and the use of surfactants [21].

On the other hand, Kopac et al. reported the effect of the quaternization reaction, its optimisation and fibril characteristic effects on the rheology of cationic micro- and nanofibrillated cellulose [22]. Moberg et al. studied the effects of particle/fibril dimensions and surface characteristics on the rheological properties of nanocellulose suspensions [23]. They have concluded that the length (or aspect ratio) of the particles played a very important role in the rheology of the suspensions.

However, no studies have been reported comparing the effect of different pre-treatments during the CNF preparation and different contents of charged groups, on the rheology and gelation of CNF suspensions. In the present work, four different samples of negatively charged CNFs (TEMPO-oxidised), containing different levels of carboxylic groups and degrees of fibrillation, were compared for their rheological behaviour with five samples of cationic CNFs (one prepared by direct cationization and four by two-step cationization) containing different levels of cationic groups and degrees of fibrillation. Additionally, dynamic light scattering and zeta potential determinations were performed to shed light on the aggregation extension and its impact on the gelation of CNF suspensions.

## 2. Results and Discussion

The type of pre-treatment during CNF production has a great impact on the properties of the obtained materials. For example, if no pre-treatment is used prior to mechanical fibrillation, the extension of nanofibre production is very limited. Moreover, the enzymatic pre-treatment can increase the degree of fibrillation, but not to very high levels. For example, the use of an enzymatic pre-treatment, followed by two HPH passes, was able to increase the degree of fibrillation from ca. 6% to ca. 17% for bleached kraft *Eucalyptus* kraft pulp [3,24]. On the other hand, the use of chemical pre-treatments enables us to obtain CNF suspensions with high degrees of fibrillation, up to 95% or more, using the same two passes in HPH. Table 1 summarises the main properties of the prepared CNFs by applying different chemical pre-treatments, followed by two passes in HPH, using as the starting material bleached *Eucalyptus* kraft pulp (BEKP).

**Figure 1:**
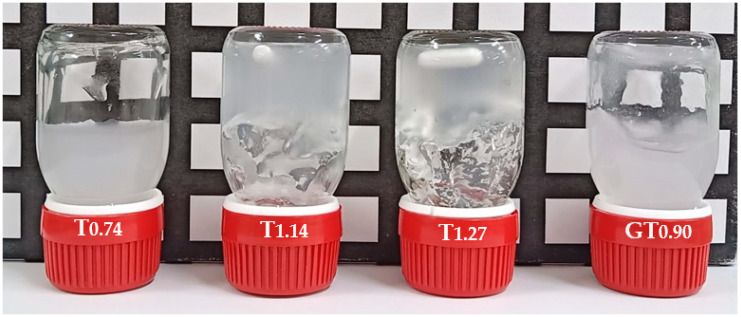
Digital photos of 0.80 wt.% aqueous suspensions of T_0.74_, T_1.14_, T_1.27_, and Cat. CNFs. The photos were taken at room temperature.

The level of nanofibrils generated by the intensive mechanical treatment (HPH) is clearly influenced by the concentration of oxidising agents, sodium hypochlorite and periodate, used during the pre-treatments. It is possible to find a clear relationship between the amount of oxidising agents and the degree of fibrillation. Increasing oxidising agents led to higher charge densities (samples T_1.41_ and GT_1.68_), resulting in the swelling of the fibres, and consequently, higher contents of nanofibrils were formed. However, these exhaustive chemical pre-treatments resulted in a relevant decrease in the degree of polymerization of cellulose molecules. For example, the starting material (BEKP) presented a degree of polymerization (DP) of ca. 2990, the sample prepared using only mechanical treatment (Mec) showed a slight decrease in DP to ca. 2300, and the sample T_1.41_ showed a DP of ca. 365, the decrease being more pronounced when periodate oxidation followed by cationization with GT reagent was used (GT_1.68_ showed a DP of only ca. 26). As a consequence, the dimensions and shape of the formed nanofibrils are also highly affected, as the fibrils formed in the sample GT_1.68_ are very short in length. One of the most valuable characteristics of CNFs is their high aspect ratio, which endows these materials with peculiar properties. One of these properties is the ability of materials with a high aspect ratio to form gels at low concentrations due to the easy network formation caused by the entanglement of the thin and long fibrils. Figure 1 presents digital photos of CNF samples with different charged groups and different contents.

From Figure 1, it is possible to infer that the degree of fibrillation has a deep impact on the ability of suspensions of cellulose nanofibres to form gels; similarly, the aspect ratio of the fibrils has a key role in their formation of gels. The sample T_0.74_ presents a moderate level of fibrillation, reducing the possibility of the formation of a strong physical network at a concentration of 0.80 wt.%, and thus a gel was not obtained at the higher concentration tested. On the other hand, the samples with higher degrees of fibrillation (T_1.14_, T_1.27_, and T_1.41_) formed gel at the same concentration due to the higher content of fibrils with the capacity to contribute to the formation of the three-dimensional network and an increased capacity to resist flow. The sample GT_0.90_ presents a fibrillation degree of ca. 98%, and thus, it should be expected to observe a strong gel for this suspension. However, due to the high destruction of the fibrils’ integrity during the pre-treatment step, the formed nanofibrils present a short length and are not prone to form a network, preventing the formation of a gel at all the tested concentrations. Long fibrils could touch each other and easily form a strong network; on the contrary, short fibrils could not touch, and the formation of highly viscous suspensions and/or gels could not be observed or was only observed for highly concentrated samples. The behaviour shown by the suspensions of CNFs follows the one observed for other polymers, where there was clearly demonstrated a high decrease in overlap concentration of the polymer as the MW increased [25].

**Figure 2:**
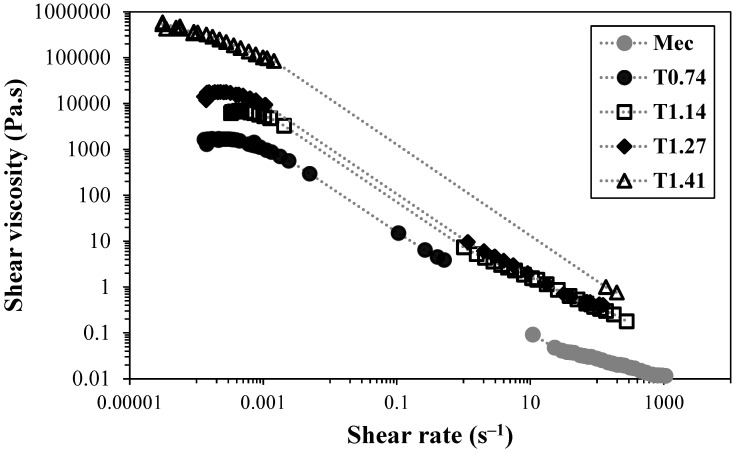
Flow curves of TEMPO-oxidised CNFs at a concentration of 0.80 wt.%, at 25 °C.

Figure 2 presents the flow curves of suspensions of TEMPO-oxidised CNFs, at ca. 0.80 wt.% and neutral pH.

The increase in the amount of sodium hypochlorite used during the pre-treatment of TEMPO-oxidised samples led to higher fibrillation of the cellulose fibres, which resulted in higher viscosity of the samples (at low shear rate values). The trend obtained shows an increase in shear viscosity of the samples at low shear rates, as well as a shift to higher stress values for the yield stress of the samples, from ca. 1 Pa for the sample with a lower charge density to ca. 100 Pa for the sample with a higher charge density. Undoubtedly, the increase in nanofibres with low thickness led to a strong reinforcement of the network, and the resistance to flow was increased about 100 times (at the same suspension concentration), due to the larger opportunity for the nanofibrils to physically interact with the neighbouring ones and form highly entangled networks, which present high resistance to flow.

**Figure 3:**
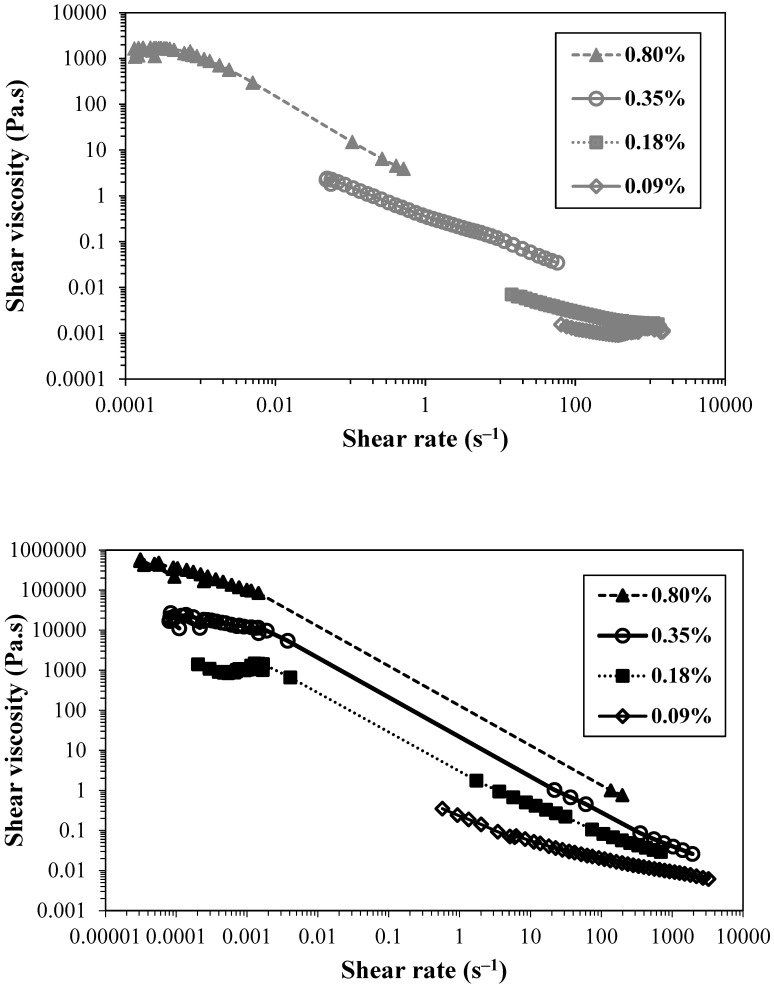
Flow curves of TEMPO-oxidised CNFs at different concentrations. (**top**)—T_0.74_; (**bottom**)—T_1.41_. The measurements were performed at 25 °C.

Independently of the CNF nanofiber content, the increase in the concentration of the TEMPO-oxidised CNF suspensions always led to an increase in the viscosity of the suspensions. Conversely, the sample without chemical pre-treatment (Mec) did not show a significant increase in viscosity, which could be attributed to the low degree of fibrillation, of only ca. 6%, whereas the TEMPO-oxidised samples always have a value equal to or above 50%. Thus, even though it could be anticipated from the intrinsic viscosity values that the formed fibrils, employing only mechanical treatment, do not present a short length, the small number of fibrils formed is not enough to increase the sample viscosity. The non-fibrillated cellulose fibres are not able to form networks at the studied concentrations but instead are prone to settle, which explains the low viscosity of the Mec CNF sample at the same concentration as the TEMPO-oxidised CNF samples. In the literature, it is possible to find works reporting gels obtained using cellulose nano/microfibres prepared by only mechanical treatment [26]. However, the authors did not report the type and intensity of mechanical treatment used, and the level of fibrillation in the sample was not reported. Nevertheless, as the used sample in the reported work is a commercial sample, it was possible to infer that the reported sample (Celish KY100S) presents a level of fibrillation of about 38% [27], which is much higher than that obtained using our method. Perhaps the difference in the level of fibrillation is related to a more intensive mechanical treatment, but in our study, we wanted to understand the effect of the chemical pre-treatment on the rheology of CNF suspensions, and thus, the mechanical treatment used was the same for all the samples (two passes only). Figure 3 shows the flow curves of TEMPO-oxidised CNFs with lower (T_0.74_) and higher (T_1.41_) charge densities.

As expected, the viscosity of the suspensions increased when the suspension concentration was raised, both for the less-charged CNFs and for the more charged ones. However, the increment in viscosity values was not of the same magnitude. For the CNF with the lowest charge, the increment in the viscosity was about six orders of magnitude, and for the highly charged CNF sample, the increment was ca. eight orders of magnitude. The presence of xylan in the starting cellulose material (14–19 wt.%) also has an impact on the rheology of the samples [28]. First, the presence of xylan decreases the oxidation kinetics, requiring longer reaction times to obtain the oxidised cellulose fibres, and second, its presence induces swelling of the cellulose particles, forming more viscous suspensions in the presence of higher contents of xylan. However, our samples were all produced employing the same BEKP batch, and thus the effect of xylan could be minimised due to the presence of an equal amount in all the samples. Similarly to the viscosity trend, the overlap concentration (transition from a diluted to a semi-diluted regime) occurs in lower concentration values for the sample with higher charge density due to the larger amount of thin and long nanofibres able to form a physical network at lower concentrations, between 0.09 and 0.18 wt.%. On the other hand, the sample with a lower charge density only attains similar values of viscosity for the higher concentration, and the overlap concentration is estimated to be between 0.18 and 0.35 wt.%, where a significant increase in viscosity is observed due to the beginning of CNF entanglements and consequent viscosity rise.

**Figure 4:**
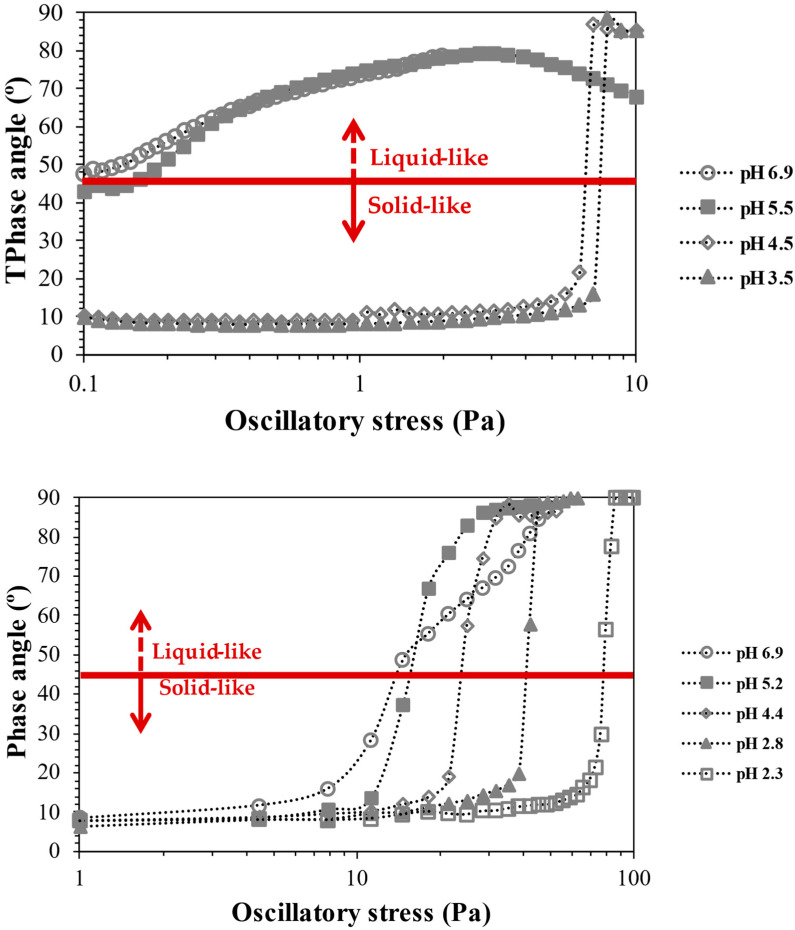
Yield stress of TEMPO CNF suspensions as function of the pH for T_0.74_ (**top**) and T_1.41_ (**bottom**). The determinations were made at 25 °C, being constant the concentration of the suspensions (0.35 wt.%).

Being the TEMPO-oxidised CNFs negatively charged, by the presence of carboxylic groups introduced in the carbon C_6_ of cellulose molecules through the sodium hypochlorite oxidation, mediated by the TEMPO radical, the behaviour of the CNFs in suspension will be deeply affected by the protonation/deprotonation of the carboxylic groups present. Figure 4 depicts the effect of the change in suspension pH, and consequently, the change in carboxylic group protonation when pH is decreased, on the yield stress of the CNF suspensions.

The decrease in the pH of the suspensions and the consequent decrease in the negative charge of CNFs increased the CNF aggregation and led to a shift in the yield stress of the suspensions. For example, for the suspension of T_0.74_, the yield stress value goes from below 0.1 Pa (below the minimum stress studied) to ca. 8 Pa with the decrease in the pH from 6.9 to 3.5. A further decrease in the pH of the suspensions of T_0.74_ resulted in the precipitation of the respective CNF due to the very low negative charge and extensive aggregation. The large decrease in negative charges by protonation of carboxylic groups led to a large loss of counterion entropy, resulting in extensive aggregation and low dispersibility of the nanofibrils, and consequent precipitation. Contrary, for the highly charged CNF, the decrease in pH of the suspension, until pH 2.3, did not result in CNF precipitation. It was observed that the effect of pH decrease is more progressive, and the larger shift in yield stress values only occurs for the transition from pH 2.8 to pH 2.3, contrary to the suspension of T_0.74_, where the main change happens for the pH transition from 5.5 to 4.5. This could be explained by the higher number of negative sites in the T_1.41_ sample, which, even facing a decrease in negative charges by lowering the pH, still presents a high enough number to avoid extensive aggregation of the nanofibrils. The larger effect observed for the sample with a lower charge density occurs close to the pKa of carboxylic groups (ca. 4.8 [29]), with approximately 50% of the carboxylic groups being protonated at this pH value. Due to the low substituent group content, this 50% decrease in charged groups is enough to give rise to extensive aggregation, and a further decrease in pH led to extensive aggregation and precipitation of cellulose particles.

**Figure 5:**
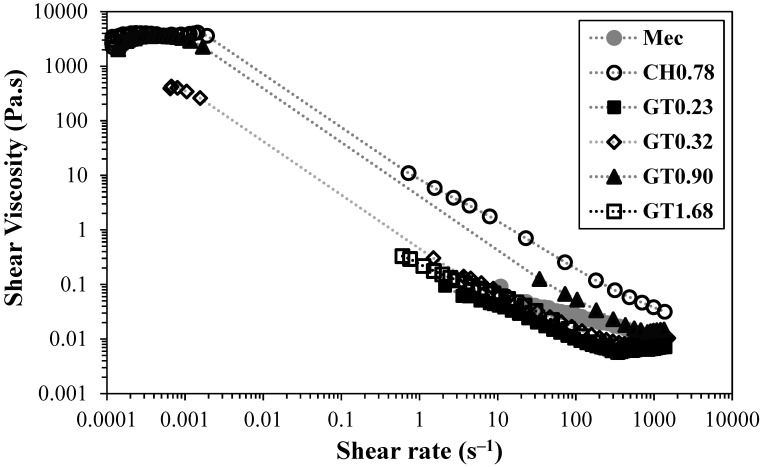
Flow curves of cationic CNFs prepared by different pre-treatments. CH_0.78_ was prepared by direct cationization with CHPTAC, and the GT series was prepared by two-step cationization. The flow curves were obtained at 25 °C for a CNF concentration of 0.80 wt.%.

Contrary to the negatively charged CNFs produced in the present work, which contain charged groups without permanent charge, the cationic ones prepared contain quaternary ammonium groups (permanent charges), and thus the amount of charged groups in the latter CNFs does not suffer the effects of pH changes. Figure 5 illustrates the flow curves of different cationic CNFs at a concentration of 0.80 wt.%.

**Figure 6:**
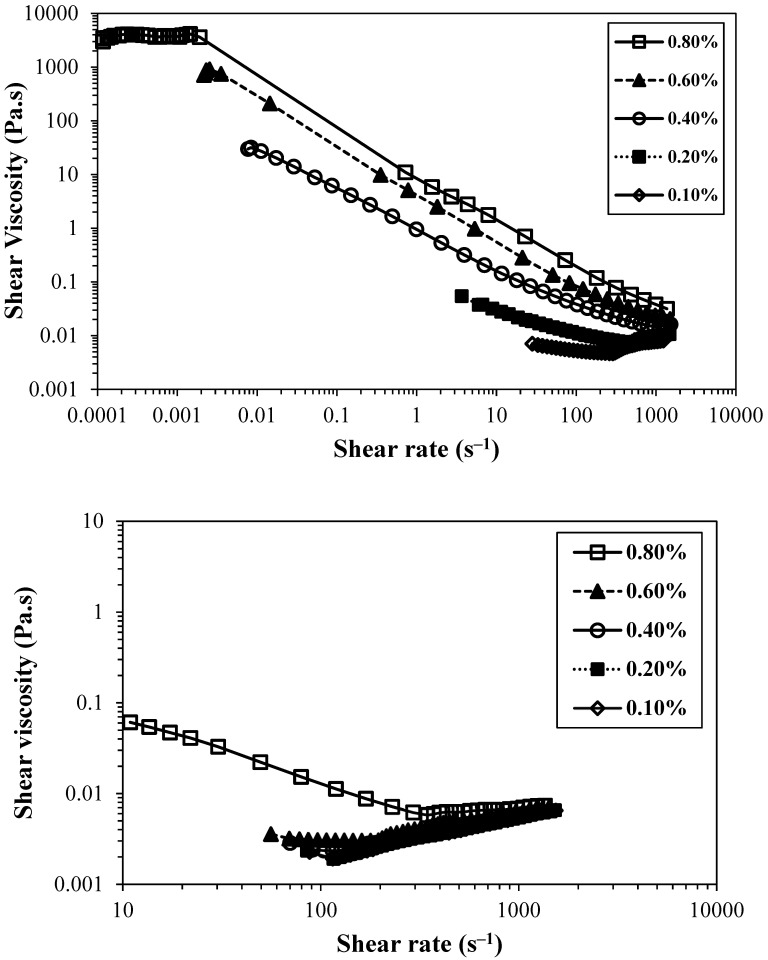
Flow curves of cationic CNFs at different concentrations. (**top**)—pre-treated with CHPTAC (CH_0.78_); (**bottom**)—pre-treated with periodate and GT reagent (GT_1.68_). Measurements were performed at 25 °C.

The sample CH_0.78_, prepared by direct cationization of cellulose with CHPTAC, even presenting a moderate degree of substitution and fibrillation, was the one that showed the highest value for yield stress point, ca. 10 Pa, of all the cationic CNF suspensions tested (obtained from viscosity vs shear stress curves). Comparing this sample with a negatively charged CNF sample with a similar charge density (T_0.74_), it is possible to observe that the cationic CNF suspension presents higher viscosity and yield stress. It is also important to note that sample T_0.74_ presents a higher degree of fibrillation (ca. 50%), almost twice the value obtained for CH_0.78_ (29%). A possible explanation for the higher viscosity obtained for a sample with lower fibrillation could be the low degradation of the cellulose molecules when CHPTAC is used for cellulose cationization and CNF preparation, as the DP obtained is very close to that of native BEKP. Thus, the formed fibrils, even if not at a very high content, are long and could easily entangle with others and form a strong network. This assumption is confirmed by the long fibrils observed in electron microscopy images obtained for the sample CH_0.78_ (Figure S1, Supplementary Material). In addition, for the samples prepared by two-step cationization, a different trend was observed compared to TEMPO-oxidised CNFs. Samples with a low degree of substitution (GT_0.23_ and GT_0.32_) presented a very low degree of fibrillation (6 and 12%, respectively), and did not form a gel, therefore presenting low-to-moderate viscosity values. The same rheological behaviour was found in the CNF sample prepared by applying only mechanical treatment due to the lower degree of fibrillation that was also obtained for this sample. In the GT series, the sample with an intermediate level of cationization (GT_0.90_) showed higher values of viscosity, which can be attributed to the high level of fibrillation obtained (98%). Making a comparison with a TEMPO-oxidised CNF sample with a similar degree of fibrillation (T_1.41_), the viscosity of the cationic CNF suspension is much lower due to the greater destruction of cellulose structure induced by the intensive oxidation during the aldehyde formation; the DP of the sample GT_0.90_ is only 47 (64 times lower than the value obtained for the starting cellulose—BEKP presents a DP of 2990); conversely, the DP of the sample T_1.41_ is only eight times lower than the DP observed for starting cellulose. Figure 6 depicts the flow curves of CH_0.78_ and cationic CNF with the highest degree of substitution for different CNF concentrations.

The difference between the samples is substantial, as the sample GT_1.68_ is very much less viscous than CH_0.78_, even though the sample prepared by direct cationization (CH_0.78_) presents a lower level of cationic groups and fibrillation degree. For samples obtained by direct cationization of cellulose hydroxyl groups, it was not possible to obtain higher degrees of cationization, even increasing the ratio cationizing agent/cellulose, due to some steric hindrance related to the position of the cellulose ring where the reaction occurs, carbon C_6_ [8,30]. Conversely, for the two-step cationization, it was possible to obtain high levels of cationization (up to 1.68 mmol/g), but an extensive degradation of the cellulose structure was observed. This destruction led to a shortening of the nanofibrils’ length and resulted in a large increment in the overlap concentration (c*). The estimated value of c* for the different cationic samples prepared was quite different, varying from 0.13 wt.% (CH_0.78_) to 0.60 wt.% (GT_1.68_), and there was also a clear influence on the viscosity of the suspensions, as observed in Figure 6. Table 2 summarizes the obtained values for the c* and critical shear rate for suspensions of the different CNFs, as well as the fitting parameters of the flow curves of the different suspensions applying the Ostwald–de Waele model. Most of the fitted curves presented a pseudoplastic behaviour (*n* < 1), and the suspensions of GT_1.68_ with concentrations below 0.8 wt.% are approximately Newtonian (*n* ≈ 1), which can be attributed to the extensive degradation of cellulose chains, resulting in short particles (in length), and in the absence of a network.

**Figure 7:**
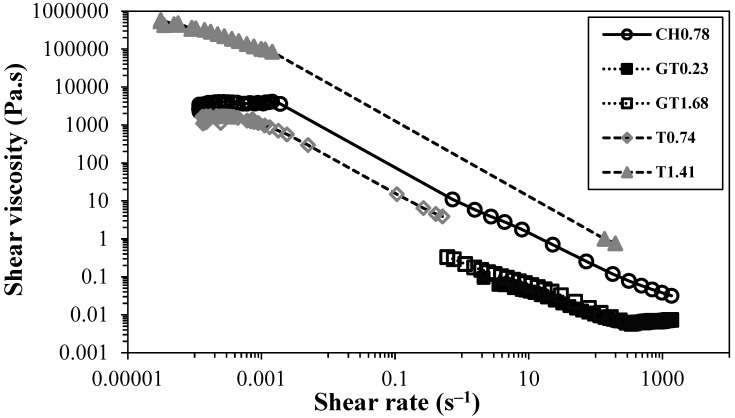
Effect of pre-treatment on the viscosity of 0.80 wt.% CNF suspensions (at 25 °C).

Figure 7 represents the flow curves of CNF suspensions prepared by applying different pre-treatments for different levels of treatment.

From Figure 7, it is clear that the pre-treatment applied, as well as the level of treatment, have a deep impact on the rheology of the CNF suspensions. Different trends were observed depending on the pre-treatment. For example, the oxidation of cellulose fibres, mediated by the TEMPO radical (samples T_0.74_ and T_1.41_), led to an increase in the viscosity of the CNF suspensions and yield stress points due to the higher level of nanofibrils produced without a drastic destruction of the cellulose structure. Contrarily, the two-step cationization (samples GT_0.90_ and GT_1.68_) resulted in extensive degradation of the cellulose structure, as can be inferred by the low intrinsic viscosity values and confirmed by observation of the fibrils formed using electron microscopy, which revealed very short particles compared to the sample CH_0.78_ (Figure S1, Supplementary Material). In addition, the presence of higher levels of cationic groups, which results in higher charge repulsion, combined with the short fibrils formed, impairs gel formation or even a suspension with high viscosity. The length of nanofibrils is demonstrated to be a critical parameter for the formation of a 3D network and induces a high increase in viscosity. Long fibrils can easily entangle and form a robust network, which results in strong resistance to flow (sample CH_0.78_). Thus, the level of nanofibrils formed after HPH plays a central role in the gelation of aqueous suspensions of charged CNF, but it was shown that this only happens if the level of shortening of CNF is not very high.

**Figure 8:**
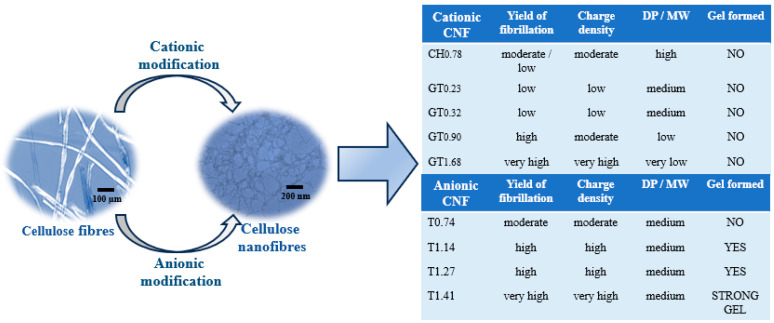
Scheme of the factors affecting viscosity and gel formation (at a CNF concentration of 0.80 wt.%) for different CNFs.

Figure 8 summarises the main properties of the CNF suspensions prepared and their influence on the rheology, referring to the formation or not of gels for suspensions containing 0.80 wt.% CNF in an aqueous medium.

## 3. Conclusions

The present work explored the effects of different pre-treatments used in the preparation of CNFs on the rheology of aqueous suspensions of CNFs containing different charged groups and different levels of modification. It was observed that TEMPO-oxidised CNFs can easily form strong and stiff gels, due to high levels of nanofibrillation and reasonable preservation of the length of the produced nanofibres. On the other hand, cationization of cellulose through a two-step procedure induces extensive degradation of the CNF structure, leading to short nanofibres and cellulose chains with low DP. As a consequence, the suspensions of cationic CNF with high cationic charge density, GT_1.68_, did not form a gel at the highest concentration studied (0.80 wt.%), and this CNF was not even able to induce a significant increase in the viscosity of the suspensions. The direct cationization method, through CHPTAC grafting on the C_6_ of cellulose molecules, generated interesting results, enabling a moderate level of nanofibrillation without extensive structure degradation. The suspensions presented high viscosity but without the formation of a gel at 0.80 wt.%. From the obtained results, it was possible to conclude that a high level of nanofibrillation combined with a not-too-low length of the obtained nanofibres are the key factors in forming strong gels. The TEMPO CNFs could fulfil these requisites, thus forming gels, whereas the cationic CNFs did not. The charge repulsion between charged groups of nanofibrils and the associated entropy of the counterions were revealed to play a role in the rheology of the suspensions, but not as pronounced as the structure of the nanofibres. The content of charged groups was revealed to be very important in keeping the nanofibres in suspension and avoiding extensive aggregation and precipitation of nanofibers when factors, such as the pH value in TEMPO-oxidised CNF suspensions, are modified and decrease the extent of those groups present in the CNF particles. In sum, the present work demonstrates the potential of the use of charged CNFs as rheology modifiers, being especially attractive to the TEMPO-oxidised CNFs with high levels of carboxylic groups, which can form gels at low concentrations. On the other hand, cationic CNFs produced by direct cationization or by two-step cationization with moderate levels of derivatization also present good rheological properties and additionally present other interesting properties such as potential antimicrobial activity. Comparing the rheological performance of the cationic and anionic modified CNFs with neutral ones, it is possible to conclude that the modification (chemical pre-treatment) has a deep impact on improving the rheological properties of CNFs. It is possible to attribute this improvement, mainly, to the better fibrillation induced by the introduction of charges on the cellulose chains.

## 4. Materials and Methods

### 4.1. Materials

The starting cellulose material used was industrial bleached *Eucalyptus* kraft pulp (BEKP), never dried, with a composition of 80–85 wt.% cellulose, 14–19 wt.% xylan, 0.3 wt.% lignin, and 0.4 wt.% extractives [31]. For the chemical surface modification of BEKP, different chemicals were used. TEMPO-mediated oxidation was carried out using NaClO (different ratios of NaClO/g of dry pulp, from 4 to 12 mmol of NaClO per g of dry pulp) and catalytic amounts of TEMPO radical and NaBr, following the methodology developed by Saito et al. [32]. 2,2,6,6-tetramethylpiperidine 1-oxyl (TEMPO radical, >98% purity) and NaBr were acquired from Sigma-Aldrich. NaClO (14% active chlorine in aqueous solution) was obtained from VWR chemicals. It was also used NaOH (pellets, acquired from VWR, Atlanta, GA, USA) to control the pH of the reaction. The cationization of the cellulose fibres was achieved by two different methods: direct cationization of cellulose using (3-chloro-2-hydroxypropyl) trimethylammonium chloride (CHPTAC) [8] and two-step cationization: oxidation with sodium periodate followed by reaction of the aldehyde groups with (carboxymethyl)trimethylammonium chloride hydrazide, Girard’s reagent T–GT [30]. For the direct cationization, CHPTAC (60 wt.% aqueous solution from Sigma Aldrich, St. Louis, MO, USA) and sodium hydroxide pellets were used during the preparation of cationic cellulose fibres. In the two-step cationization, sodium periodate (NaIO_4_, from SP, Cleveland, OH, USA), acetic acid glacial (from VWR), isopropanol (from Labsolve), and GT (from Sigma-Aldrich) were used. For the determination of the content in aldehyde groups and charge density (cationic and anionic groups), hydroxylamine hydrochloride, sodium hydroxide, and hydrochloric acid 1 M aqueous solutions (all from Sigma-Aldrich) were used in the titrations. Distilled water was used for the preparation of all the samples. All chemicals were used as received without further purification.

### 4.2. Chemical Modification (Pre-Treatment) and Preparation of Nanofibres

Mec CNF: A sample of CNF without any chemical pre-treatment was prepared using only mechanical treatment. To do so, the cellulose fibres were dispersed at 1.0 wt.% consistency in distilled water and mechanically treated in a high-pressure homogeniser (GEA Niro Soavi, model Panther NS3006 L, GEA Mechanical Equipment, Parma, Italy), using two passes through the homogeniser, first at 500 bar and next at 1000 bar. A suspension with a consistency of 0.96 wt.% was obtained.

TEMPO-mediated oxidation: To prepare TEMPO CNF samples, the cellulose fibres (industrial never-dried bleached *Eucalyptus globulus* kraft pulp) were dispersed in water, being previously dissolved in water the necessary amounts of TEMPO radical and NaBr (0.016 g of TEMPO and 0.1 g of NaBr per g of dry pulp). Afterwards, the fibres were oxidised with NaClO using different ratios (from 4 to 12 mmol of NaClO per g of dry pulp). The reactions were conducted for two hours (at room temperature) until the pH of the suspension remained stable at a pH value of 10. The oxidised fibres were filtered and washed thoroughly with distilled water, until the washing water conductivity reached a constant value, close to the value of dis-tilled water. Finally, the oxidised fibres were dispersed at ca. 1.0 wt.% consistency in distilled water and mechanically treated in a high-pressure homogeniser (GEA Niro Soavi, model Panther NS3006 L), using two passes through the homogeniser, first at 500 bar and next at 1000 bar. Suspensions with a consistency in the range of 0.80 and 1.00 wt.% were obtained.

Direct cationization: a direct cationization method was used to covalently bond quaternary ammonium groups to BEKP using CHPTAC, a precursor of 2,3-epoxypropyltrimethylammonium chloride (EPTAC) [11]. The reactive epoxide was prepared in situ by alkalinization of CHPTAC with sodium hydroxide [8]. The formed epoxide reacted with the available hydroxyl groups of cellulose, in alkaline conditions, to form an ether linkage at the C_6_ position of the anhydroglucose unit (AGU), adding cationic charges to cellulose. BEKP was dispersed at 3% consistency in water containing NaOH (at a molar ratio of NaOH/AGU of 9). The fibre suspension was stirred for 20 min at 20 °C, after which CHPTAC was added in a CHPTAC/AGU molar ratio of 6 [8]. The reaction followed at 65 °C for 8 h. Then, the obtained cationized fibres were filtered and washed thoroughly with distilled water until the washing water conductivity reached a constant value, close to the value of distilled water. Finally, the pre-treated cationic fibres were dispersed in distilled water at ca. 1.0 wt.% consistency and mechanically treated in a high-pressure homogeniser (GEA Niro Soavi, model Panther NS3006 L), also applying two passes through the homogeniser, first at 500 bar and second at 1000 bar. Suspensions containing 0.90 wt.% of cationic cellulose nanofibres (CH_0.78_) were obtained.

Two-step cationization: the second method to add cationic charges to cellulose consisted of indirect cationization (two-step). To do so, first cellulose was converted into a more reactive form, dialdehyde cellulose (DAC), through oxidation with sodium metaperiodate (NaIO_4_). This reaction opened the C_2_–C_3_ bond and converted the two vicinal hydroxyl groups of AGU into aldehyde groups [26]. Cellulose cationization was attained by reacting the DAC with the GT reagent, obtaining a stable imine structure with quaternary ammonium groups [33]. The first step of the reaction was the preparation of DAC, which was performed by dispersing BEKP in a water/isopropanol (IPA) mixture (10% *v*/*v* IPA) to act as a free radical scavenger and reduce the depolymerization of cellulose [34] at 70 °C with a consistency of 2.5 wt.% of cellulose fibres. Different molar ratios of sodium periodate/AGU were used to obtain different levels of cellulose oxidation, ranging from 0.10 to 0.65 mol of sodium periodate per mol of AGU. The reactions were left to continue for 4 h in the absence of light to minimise the photodegradation of sodium periodate. Afterwards, the oxidised fibres (DACs) were extensively washed with water, and the content in aldehyde groups (degree of substitution, DSa) was determined based on the oxime reaction between the aldehyde groups and hydroxylamine hydrochloride by potentiometric titration (using sodium hydroxide as titrating agent [35]). After determining the number of aldehyde groups in each sample, the undried DACs were dispersed in acidified water (10% *v*/*v* of acetic acid) at a consistency of 5.0 wt.%. Based on the previous determination of aldehyde groups, a specific molar ratio of GT to aldehyde groups was loaded into the reaction media, ranging from 0.6 to 1.0 mol of GT per mol of aldehyde [8]. The reaction between the GT reagent and the aldehyde groups of cellulose was performed at 70 ◦C for 90 min. Then, the samples were filtered, under vacuum, through a filter paper with a pore size of 11 μm and extensively washed with a mixture of water/IPA (1:9 *v*/*v*), until the conductivity of the washing solvent was constant. The cationic cellulose fibres were dispersed in water (1.0 wt.%) and subjected to a two-pass mechanical treatment in an HPH (GEA Niro Soavi, model Panther NS3006L), with a first pass at 500 bar and a second pass at 1000 bar. Suspensions with a consistency varying between 0.90 and 1.10 wt.% were obtained.

### 4.3. Characterization of the Obtained Cellulose Nanofibres

For all the prepared samples, the degree of fibrillation, degree of polymerization, zeta potential, hydrodynamic radius, and charge density were measured. The degree of nanofibrillation was determined in duplicate after centrifugation of aqueous CNF suspensions (0.2 wt.%) at 9000 rpm for 30 min. The precipitate was discarded, and the content of the supernatant (after water removal) was assumed to be the content of the sample in nanofibrillated material. For determination of the degree of polymerization, the CNF samples were dissolved in cupriethylenediamine, and the intrinsic viscosity was determined according to ISO standard 5351:2010. The degree of polymerization (DP) was then estimated using the Mark-Houwink equation, with parameters K = 0.42, a = 1 for DP < 950 and K = 2.28, a = 0.76 for DP > 950 [36].

The hydrodynamic radius of the cellulose nanofibrils was obtained by dynamic light scattering measurements. The experiments were carried out at 25 °C in Zetasizer NanoZS equipment (ZN 3500, Malvern Instruments, Malvern, UK), with a laser of 532 nm wavelength, using a backscatter angle detection of 173°. The concentration of CNF suspensions used was 0.05 wt.%, being the suspensions first centrifuged at 4000 rpm for 10 min, previously to size determinations, in order to remove residual large particles present in the suspension. The concentration of the suspensions used for size determination was lower than the estimated overlap concentration (for all the prepared samples) to avoid extensive interactions among nanofibrils. A glass cuvette was used for all the size determinations, with the suspensions gently inserted in the cuvette and checked for the presence of bubbles. The particle size was determined, based on six repetitions, according to the non-negative least-squares (NNLS) algorithm using the Zetasizer Nano software (version 7.11). Additionally, zeta potential measurements were carried out in the same equipment, using appropriate zeta cuvettes, and the zeta potential values were estimated using the same software, by electrophoretic light scattering, based on six repetitions for each sample.

The charge density of the different CNF samples was estimated by titration, according to the nature of the sample. For TEMPO-oxidised samples, the carboxyl content was determined, in duplicate, by conductometric titration of aqueous suspensions of CNFs (acidified to a pH of ca. 3 with HCl) and titrated with NaOH 0.01 M (standard solution). The charge density (mmol/g) corresponds to the CNF sample carboxyl content. Moreover, the charge density of cationic CNF samples (also expressed in mmol/g) was estimated using a potentiometric titration method [37]. To do so, a given mass of the different cationic CNF suspensions (corresponding to 200 mg dry matter) was dispersed in 150 mL of distilled water, and the pH of the suspension was adjusted to 11 using a 0.1 M NaOH aqueous solution (this step allows the conversion of the cationic quaternary ammonium groups to their OH^−^ form by exchanging the Cl^−^ with OH^−^ ions). The suspensions were then titrated at a temperature of 35 °C using 0.01 M HCl (standard solution) until the inflexion point of the pH curve was attained.

### 4.4. Rheology of CNF Suspensions

#### 4.4.1. Preparation of Nanofibre Suspensions

The CNF suspensions for rheology studies were prepared by dilution of the original suspensions of nanofibres, by weighing the desired amount of suspension and adding the required mass of distilled water. Suspensions of different concentrations from 0.80 to 0.09 wt.% of the different CNFs containing different charged groups and at different levels of modification were prepared. Suspensions were kept under stirring for 30 min to ensure a good dispersion of the nanofibres. To study the effect of the pH of the suspension, a 0.5 M stock solution of acetic acid was prepared, and the pH of the suspensions was adjusted to the desired values, between neutral pH and pH of ca. 2.3. After the pH adjustment, all the samples were left to equilibrate for 30 min under agitation, and the pH was measured again after this period to check for possible variations.

#### 4.4.2. Rheology of the Suspensions

All the rheological studies were performed at constant temperature (25 °C), ensured by a water recirculatory bath (Haake Phoenix II, Karlsruhe, Germany), in a controlled stress rheometer (Haake, Model RS1, Karlsruhe, Germany), using different geometries. A cone-plate geometry (C60/1) was used for the rotational tests of TEMPO-oxidised CNF suspensions and a plate-plate geometry (PP20Ti) for the oscillatory tests. For the suspensions of cationic CNFs, a cylindrical bob and cup sensor (Z34 DIN Ti) with a gap of 7.2 mm was used, due to the low viscosity of some suspensions. For all the samples, flow curves were obtained in controlled stress mode by applying shear stresses ranging between 0.1 and 150.0 Pa, with the range adjusted to each sample for better data acquisition. For each flow curve, 40 steps were acquired, with a duration of 30 s for each step and an integration time of 15 s. The rheological response of the flow tests can be modelled by the power law Ostwald-de-Waele equation [13], written as:

$$\tau =K{\dot{\gamma }}^{n}$$

where *τ* is the shear stress (in Pa), *K* is the consistency index (in (Pa s^n^), $\dot{\gamma }$ is the shear rate (in s^−1^) and *n* is the flow behaviour index. A *n* equal to 1 reveals a Newtonian fluid. Non-Newtonian fluids are indicated by *n* values different from unity; a n lower than 1 indicates a pseudoplastic (or shear thinning) behaviour, whereas a *n* higher than 1 indicates a dilatant (or shear thickening) behaviour. The phase angle dependence on the applied oscillatory stress was accessed by performing stress sweep oscillatory experiments from 0.1 to 100 Pa, with the range adapted to each sample. Yield stress points were obtained from oscillation amplitude sweep tests (cross-over of G′/G″). The changes in the structure of suspensions are given by the oscillatory stress dependence of the phase angle (δ), which describes the phase lag between the viscous and elastic components of the complex modulus, where:

$$\tan\delta =\frac{G^{\prime \prime }}{G^{\prime }}$$

Values of δ above 45° indicate tan δ > 1 and G″ > G′ (predominantly viscous behaviour), while values of δ below 45° indicate G′ > G″ (predominantly elastic behaviour), and δ = 45° indicates the phase transition (G″ = G′) [38]. Rheological data were treated using the Haake RheoWin 4.20.005 software (Haake, Vreden, Germany).

## Figures and Tables

**Table 1:** Characterization of the produced CNFs.

CNF	Charge Density (mmol/g)	Degree of Fibrillation (%)	Intrinsic Viscosity (mL/g)	Degree of Polymerization	Zeta Potential (mV)	Hydrodynamic Radius (nm)
Mec	0.12	6.5	815	2287	−14 ± 4	861 ± 151
CH_0.78_	0.78	29.0	965	2859	+26 ± 3	---
GT_0.23_	0.23	6.0	183	437	+24 ± 2	---
GT_0.32_	0.32	12.0	102	244	+29 ± 5	---
GT_0.90_	0.90	98.0	20	47	+27 ± 2	147 ± 4
GT_1.68_	1.68	100.0	11	26	+30 ± 3	106 ± 15
T_0.74_	0.74	49.3	160	381	−42 ± 10	381 ± 19
T_1.14_	1.14	65.5	143	340	−46 ± 4	479 ± 40
T_1.27_	1.27	88.1	150	355	−49 ± 6	317 ± 21
T_1.41_	1.41	100.0	153	365	−82 ± 10	161 ± 14

Mec—only mechanical treatment; CH—direct cationization with CHPTAC; GT—two-step cationization with Girard T reagent; and T—TEMPO-mediated oxidation.

**Table 2:** Parameters obtained for the fitting of the flow curves of the different suspensions with the Ostwald–de Waele model. *K* is the consistency index; *n* is the flow behaviour index. Also, the obtained values for C.S.R. (critical shear rate) and c* (overlap concentration).

Sample	*k* (Pa·s^n^)	*n*	r	C.S.R. (s^−1^)	c* (wt.%)
T_0.74_	0.897	0.409	0.997	6.0 × 10^−4^	0.18–0.35
T_1.14_	7.241	0.340	0.999	9.0 × 10^−4^	----
T_1.27_	796.2	0.624	0.997	5.0 × 10^−4^	----
T_1.41_	2136	0.440	0.987	3.0 × 10^−4^	0.09–0.18
T_0.74_ (1)	0.380	0.404	0.996	5.0 × 10^−2^	----
T_0.74_ (2)	0.025	0.763	0.994	----	----
T_0.74_ (3)	0.019	0.607	0.993	----	----
T_1.41_ (1)	911.6	0.632	0.993	1.0 × 10^−3^	----
T_1.41_ (2)	94.39	0.616	0.973	2.0 × 10^−3^	----
T_1.41_ (3)	0.235	0.361	0.995	----	----
T_0.74_ (1.1)	385.5	0.595	0.994	3.0 × 10^−4^	----
T_1.41_ (1.2)	144.0	0.310	0.998	2.0 × 10^−5^	----
CH_0.78_	839.6	0.733	0.992	1.8 × 10^−3^	0.13
GT_0.23_	0.171	0.402	0.998	----	0.40
GT_0.32_	12.12	0.503	0.999	8.0 × 10^−4^	0.42
GT_0.90_	95.75	0.615	0.994	1.9 × 10^−3^	0.32
GT_1.68_	0.241	0.390	0.999	----	0.60
CH_0.78_ (4)	25.16	0.360	1.000	3.5 × 10^−3^	----
CH_0.78_ (5)	0.651	0.298	1.000	9.0 × 10^−3^	----
CH_0.78_ (6)	0.061	0.625	0.997	----	----
CH_0.78_ (7)	0.015	0.768	0.992	----	----
GT_1.68_ (4)	0.005	0.998	0.957	----	----
GT_1.68_ (5)	0.004	1.170	0.970	----	----
GT_1.68_ (6)	0.003	1.027	0.955	----	----
GT_1.68_ (7)	0.002	1.048	0.967	----	----

(1) concentration = 0.35 wt.%; (2) concentration = 0.18 wt.%; (3) concentration = 0.09 wt.%; (1.1) concentration = 0.35 wt.% at a pH of 3.5; (1.2) concentration = 0.35 wt.% at a pH 2.3; (4) concentration = 0.60 wt.%; (5) concentration = 0.40 wt.%; (6) concentration = 0.20 wt.%; and (7) concentration = 0.10 wt.%.

## Data Availability

The original contributions presented in the study are included in the article/Supplementary Materials, further inquiries can be directed to the corresponding author/s.

## Supplementary Materials

The following supporting information can be downloaded at https://www.mdpi.com/article/10.3390/gels10060367/s1, Figure S1: Scanning electron microscopy images of CNF suspensions. Top: CH_0.78_; middle: GT_0.90_; bottom: GT_1.68_.

## References

[1] Djafari Petroudy S.R., Chabot B., Loranger E., Naebe M., Shojaeiarani J., Gharehkhani S., Ahvazi B., Hu J., Thomas S. (2021). Recent Advances in Cellulose Nanofibers Preparation through Energy-Efficient Approaches: A Review. Energies.

[2] Surendran G., Sherje A.P. (2022). Cellulose nanofibers and composites: An insight into basics and biomedical applications. J. Drug Deliv. Sci. Technol..

[3] Alves L., Ramos A., Rasteiro M.G., Vitorino C., Ferraz E., Ferreira P.J.T., Puertas M.L., Gamelas J.A.F. (2022). Composite Films of Nanofibrillated Cellulose with Sepiolite: Effect of Preparation Strategy. Coatings.

[4] Jeencham R., Le H.M., Numpaisal P.-O., Ruksakulpiwat Y. (2023). Preparation of cellulose nanofiber from cassava pulp by high-pressure homogenizer. Proceedings of the 37th International Conference of the Polymer Processing Society (PPS-37).

[5] Nechyporchuk O., Belgacem M.N., Bras J. (2016). Production of cellulose nanofibrils: A review of recent advances. Ind. Crops Prod..

[6] Jowkarderis L., van de Ven T.G.M. (2015). Rheology of semi-dilute suspensions of carboxylated cellulose nanofibrils. Carbohydr. Polym..

[7] Turbak A.F., Snyder F.W., Sandberg K.R. (1983). Microfibrillated Cellulose, a New Cellulose Product: Properties, Uses, and Commercial Potential.

[8] Pedrosa J.F.S., Rasteiro M.G., Neto C.P., Ferreira P.J.T. (2022). Effect of cationization pretreatment on the properties of cationic Eucalyptus micro/nanofibrillated cellulose. Int. J. Biol. Macromol..

[9] Tavakolian M., Jafari S.M., van de Ven T.G.M. (2020). A Review on Surface-Functionalized Cellulosic Nanostructures as Biocompatible Antibacterial Materials. Nano-Micro Lett..

[10] Ribau Teixeira M., Ismail A., Medronho B., Alves L., Pedrosa J.F.S., Ferreira P.J.T., Serrão Sousa V., Rosa da Costa A.M. (2024). Nanofibrillated cationic cellulose derivatives as flocculants for domestic wastewater treatment. J. Water Process Eng..

[11] Song Y., Sun Y., Zhang X., Zhou J., Zhang L. (2008). Homogeneous Quaternization of Cellulose in NaOH/Urea Aqueous Solutions as Gene Carriers. Biomacromolecules.

[12] Alves L., Lindman B., Klotz B., Böttcher A., Haake H.-M., Antunes F.E. (2015). Rheology of polyacrylate systems depends strongly on architecture. Colloid Polym. Sci..

[13] Alves L., Ferraz E., Lourenço A.F., Ferreira P.J.T., Rasteiro M.G., Gamelas J.A.F. (2020). Tuning rheology and aggregation behaviour of TEMPO-oxidised cellulose nanofibrils aqueous suspensions by addition of different acids. Carbohydr. Polym..

[14] Nechyporchuk O., Belgacem M.N., Pignon F. (2016). Current Progress in Rheology of Cellulose Nanofibril Suspensions. Biomacromolecules.

[15] Hubbe M.A., Tayeb P., Joyce M., Tyagi P., Kehoe M., Dimic-Misic K., Pal L. (2017). Rheology of Nanocellulose-rich Aqueous Suspensions: A Review. BioResources.

[16] Sato Y., Kusaka Y., Kobayashi M. (2017). Charging and Aggregation Behavior of Cellulose Nanofibers in Aqueous Solution. Langmuir.

[17] Fukuzumi H., Tanaka R., Saito T., Isogai A. (2014). Dispersion stability and aggregation behavior of TEMPO-oxidized cellulose nanofibrils in water as a function of salt addition. Cellulose.

[18] Šebenik U., Krajnc M., Alič B., Lapasin R. (2019). Ageing of aqueous TEMPO-oxidized nanofibrillated cellulose dispersions: A rheological study. Cellulose.

[19] Calabrese V., Muñoz-García J.C., Schmitt J., da Silva M.A., Scott J.L., Angulo J., Khimyak Y.Z., Edler K.J. (2019). Understanding heat driven gelation of anionic cellulose nanofibrils: Combining saturation transfer difference (STD) NMR, small angle X-ray scattering (SAXS) and rheology. J. Colloid Interface Sci..

[20] Da Silva M.A., Calabrese V., Schmitt J., Celebi D., Scott J.L., Edler K.J. (2018). Alcohol induced gelation of TEMPO-oxidized cellulose nanofibril dispersions. Soft Matter.

[21] Quennouz N., Hashmi S.M., Choi H.S., Kim J.W., Osuji C.O. (2016). Rheology of cellulose nanofibrils in the presence of surfactants. Soft Matter.

[22] Kopač T., Krajnc M., Ručigaj A. (2022). A rheological study of cationic micro- and nanofibrillated cellulose: Quaternization reaction optimization and fibril characteristic effects. Cellulose.

[23] Moberg T., Sahlin K., Yao K., Geng S., Westman G., Zhou Q., Oksman K., Rigdahl M. (2017). Rheological properties of nanocellulose suspensions: Effects of fibril/particle dimensions and surface characteristics. Cellulose.

[24] Alves L., Ramos A., Ferraz E., Ferreira P.J.T., Rasteiro M.G., Gamelas J.A.F. (2023). Design of cellulose nanofibre-based composites with high barrier properties. Cellulose.

[25] Kronberg B., Holmberg K., Lindman B. (2014). Polymers in Solution. Surface Chemistry of Surfactants and Polymers.

[26] Gorbacheva S.N., Ilyin S.O. (2021). Morphology and Rheology of Heavy Crude Oil/Water Emulsions Stabilized by Microfibrillated Cellulose. Energy Fuels.

[27] Molinari G., Gigante V., Fiori S., Aliotta L., Lazzeri A. (2021). Dispersion of Micro Fibrillated Cellulose (MFC) in Poly(lactic acid) (PLA) from Lab-Scale to Semi-Industrial Processing Using Biobased Plasticizers as Dispersing Aids. Chemistry.

[28] Pääkkönen T., Dimic-Misic K., Orelma H., Pönni R., Vuorinen T., Maloney T. (2016). Effect of xylan in hardwood pulp on the reaction rate of TEMPO-mediated oxidation and the rheology of the final nanofibrillated cellulose gel. Cellulose.

[29] Wågberg L., Decher G., Norgren M., Lindström T., Ankerfors M., Axnäs K. (2008). The Build-Up of Polyelectrolyte Multilayers of Microfibrillated Cellulose and Cationic Polyelectrolytes. Langmuir.

[30] Pedrosa J.F.S., Alves L., Neto C.P., Rasteiro M.G., Ferreira P.J.T. (2022). Assessment of the Performance of Cationic Cellulose Derivatives as Calcium Carbonate Flocculant for Papermaking. Polymers.

[31] Henriques P., Martinho M., Serrano M.d.L., Mendes de Sousa A.P., Brites Alves A.M. (2021). Xylooligosaccharides production by acid hydrolysis of an alkaline extraction filtrate from Eucalyptus globulus bleached kraft pulp. Ind. Crops Prod..

[32] Saito T., Kimura S., Nishiyama Y., Isogai A. (2007). Cellulose Nanofibers Prepared by TEMPO-Mediated Oxidation of Native Cellulose. Biomacromolecules.

[33] Grenda K., Arnold J., Gamelas J.A.F., Rasteiro M.G. (2017). Environmentally friendly cellulose-based polyelectrolytes in wastewater treatment. Water Sci. Technol..

[34] Kristiansen K.A., Potthast A., Christensen B.E. (2010). Periodate oxidation of polysaccharides for modification of chemical and physical properties. Carbohydr. Res..

[35] Wei J., Du C., Liu H., Chen Y., Yu H., Zhou Z. (2016). Preparation and Characterization of Aldehyde-Functionalized Cellulosic Fibers through Periodate Oxidization of Bamboo Pulp. BioResources.

[36] Henriksson M., Berglund L.A., Isaksson P., Lindström T., Nishino T. (2008). Cellulose Nanopaper Structures of High Toughness. Biomacromolecules.

[37] Shunkevich A.A., Akulich Z.I., Mediak G.V., Soldatov V.S. (2005). Acid–base properties of ion exchangers. III. Anion exchangers on the basis of polyacrylonitrile fiber. React. Funct. Polym..

[38] Hiroshi M., Ailton De Souza G. (2012). Rheology—Theory and Application to Biomaterials. Polymerization.

