# Supplementary material for: Rheology of Suspensions of TEMPO-Oxidised and Cationic Cellulose Nanofibrils—The Effect of Chemical Pre-Treatment
Source: Gels. 2024 May 26;10(6):367. doi: 10.3390/gels10060367 (PMC11202593; doi:10.3390/gels10060367)
Supplement: Supplementary file 1 [file gels-10-00367-s001.zip › gels-2965481-supplementary.pdf]

Supplementary material

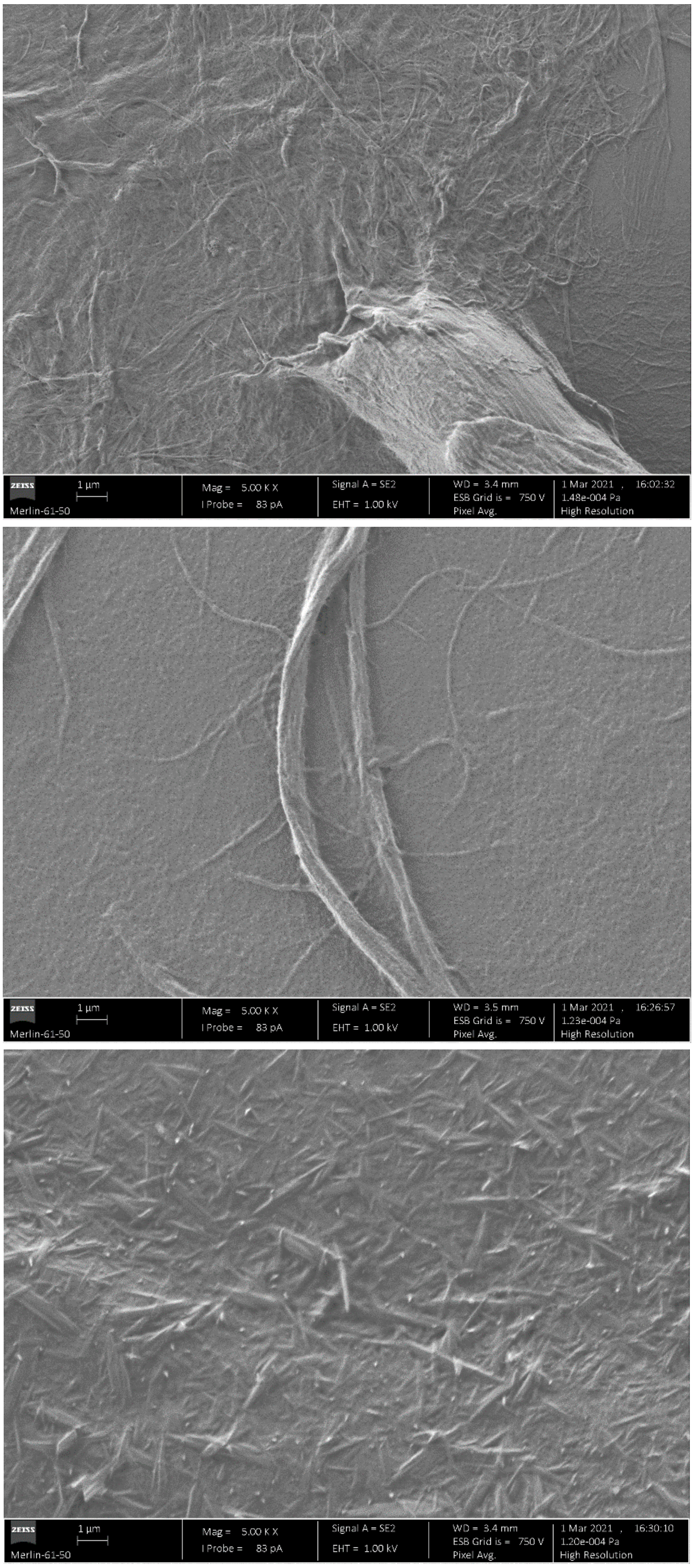

Figure S1: Scanning electron microscopy images of CNF suspensions. Top: CH0.78; middle: GT0.90; bottom: GT1.68.
